# EGCG impedes human Tau aggregation and interacts with Tau

**DOI:** 10.1038/s41598-020-69429-6

**Published:** 2020-07-28

**Authors:** Shweta Kishor Sonawane, Hariharakrishnan Chidambaram, Debjyoti Boral, Nalini Vijay Gorantla, Abhishek Ankur Balmik, Abha Dangi, Sureshkumar Ramasamy, Udaya Kiran Marelli, Subashchandrabose Chinnathambi

**Affiliations:** 1 Neurobiology Group, Division of Biochemical Sciences, CSIR-National Chemical Laboratory, Dr. Homi Bhabha Road, Pune, 411008 India; 2 Structural Biology Group, Division of Biochemical Sciences, CSIR-National Chemical Laboratory, Dr. Homi Bhabha Road, Pune, 411008 India; 3 Central NMR Facility and Division of Organic Chemistry, CSIR-National Chemical Laboratory, Dr. Homi Bhabha Road, Pune, 411008 India; 4 Academy of Scientific and Innovative Research (AcSIR), Pune, 411008 India

**Keywords:** Biochemistry, Biophysics, Chemical biology, Neuroscience

## Abstract

Tau aggregation and accumulation is a key event in the pathogenesis of Alzheimer’s disease. Inhibition of Tau aggregation is therefore a potential therapeutic strategy to ameliorate the disease. Phytochemicals are being highlighted as potential aggregation inhibitors. Epigallocatechin-3-gallate (EGCG) is an active phytochemical of green tea that has shown its potency against various diseases including aggregation inhibition of repeat Tau. The potency of EGCG in altering the PHF assembly of full-length human Tau has not been fully explored. By various biophysical and biochemical analyses like ThS fluorescence assay, MALDI-TOF analysis and Isothermal Titration Calorimetry, we demonstrate dual effect of EGCG on aggregation inhibition and disassembly of full-length Tau and their binding affinity. The IC50 for Tau aggregation by EGCG was found to be 64.2 μM.

## Introduction

**Figure 1:**
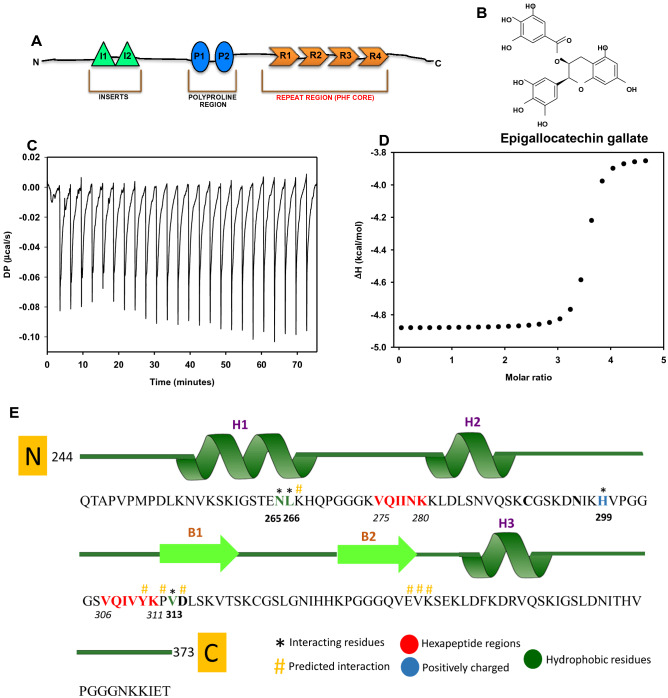
The interaction and binding affinity of Tau and EGCG. (**A**) Tau domain organization. (**B**) The structure of polyphenol EGCG. (**C**) Isothermal titration calorimetry carried out for 20 μM full-length Tau and 500 μM EGCG shows multi-site binding between the two. (**D**) The heat plot for Tau-EGCG interaction suggests n = 3.5 binding sites. Thus, there are more than one binding events involved in Tau-EGCG interaction. (**E**) A schematic diagram of the Tau K18 model depicting three predicted alpha helices (H1, H2, and H3) and two predicted beta sheets (B1, B2) with two distinct and characteristic hexa-peptide regions ^275^VQIINK^280^ and ^306^VQIVYK^311^. The predicted ligand interacting residues, color coded according to their type of interaction is also shown.

The direct causal relationship between neurodegenerative disorders and protein misfolding has been debatable over years^1–3^. The recent advances in studies of misfolded proteins to some extent have shed the light upon the cause-effect interdependence of proteins and diseases^4^. Neurodegenerative diseases are diverse in nature at physiological as well as molecular level. For example, the misfolded proteins involved in Alzheimer’s disease (AD) are amyloid-β (Aβ) and Tau (Fig. 1A) whereas^5^; α-Synuclein is a key player in Parkinson’s disease. AD is phenotypically characterized by gradual memory loss due to neuronal death^6^. The molecular pathology involves abnormal accumulation of Aβ plaques^7^ in the extracellular milieu and cytoplasmic Tau tangles^8,9^. Tau aggregation increases load on clearance machinery of neurons, which finally collapses, and results in neuronal death^10,11^. The physiological function of Tau is to bind microtubules and stabilize them thus aiding in neuronal functioning^12,13^. Tau aggregates abnormally due to multiple factors-like mutations, aberrant post-translational modifications, oxidative stress etc*.*^14–16^. Abnormal phosphorylation of Tau is one of the key factors implied in its aggregation^17,18^. The factors leading to Tau aggregation have directed the researchers to design and develop the therapeutics against Tau aggregation and AD. Several classes of compounds showing potency in inhibiting Tau aggregation include phenothiazines^19,20^, anthraquinones^21^, porphyrins, aminothienopyridazines^22^, natural compounds (polyphenols ^23^, secondary metabolites^24^, curcumin^25^, Oleocanthal^26^ etc.). The phenothiazine compounds inhibit Tau-Tau (repeat domain) binding thus preventing Tau aggregation. These compounds have a varied potency in inhibiting Tau-Tau binding. For example, Thionine and Azure A have inhibitory constant of 98 nM and 108 nM, respectively whereas chlorpromazine and tacrine have inhibitory constants 55.9 μM and > 100 μM^19^. Oleocanthal, an aglycone in extra vigin olive oil inhibits aggregation of full-length and repeat Tau. The IC50 value for repeat Tau mutant P301L (K18PL) for natural and unnatural oleocanthal was found to be 2.9 μM and 3 μM, respectively^26^.

Epigallocatechin-3-gallate (EGCG) is an active component of green tea belonging to class of polyphenols (Fig. 1B). EGCG has been implicated in preventing aggregation of proteins involved in disease conditions^27^. It inhibits aggregation of transthyretin protein involved in peripheral neuropathy^28^. Islet amyloid polypeptide (IAPP) associated with the pathology of type-2 diabetes misfolds and deposits in the β-cells of pancreas. EGCG prevents the amyloid seed formation by IAPP and also disaggregates the preformed amyloid fibrils^29,30^. In scrapie-infected cells EGCG prevented the formation of PrP^Sc^ thus showing its effectiveness against misfolding of prion protein^31^. EGCG has been studied extensively with respect to protein misfolding in AD. EGCG hampers the aggregation of Aβ by inducing off-pathway oligomers that did not stimulate fibrillogenesis. Oxidized EGCG hydrophobically remodels the amyloid fibrils decreasing the competency of oligomers formed^32^. Repeat Tau (K18ΔK280) aggregation is prevented by EGCG at low concentration and rescues the aggregate mediated toxicity of neuronal cells^33^. We show the efficacy of EGCG to prevent the aggregation and disaggregation of full-length Tau in vitro. To further explore the probable binding site, mode of binding and affinity of EGCG with Tau, ITC and molecular docking and simulation studies were done. The binding affinity of full-length Tau with EGCG was determined by ITC whereas; the docking and simulation studies were done with the modeled hexapeptide repeat region of Tau.

## Results

### EGCG interacts with Tau in a multiple binding event

Tau as a therapeutic target in AD has been explored extensively. The protein–ligand binding affinity plays a key role in determining the functional consequences at the molecular level. Full-length Tau and EGCG binding was determined by using isothermal titration calorimetry. ITC result suggests that EGCG binds to Tau at multiple sites. The binding isotherm showing the heat change over the course of titration indicates initial decrease in enthalpy followed by subsequent increase until the end point of titration in a 25 injections program (Fig. 1C). This suggested that the initial binding of EGCG is rapid and constrains the further binding with Tau. However, the negative value of free energy changes (ΔG) of the reaction indicates that EGCG binding to Tau is spontaneous and favourable (Fig. S1). The heat plot between the heat change per mole of the reactants EGCG and Tau and their molar ratio gives the number of interacting sites as 3.48 (Fig. 1D) and the Kd was determined from the thermodynamic parameters as 74 nM (Table 1). These results suggested binding between Tau and EGCG occurs in a multi-sites binding event such that the initial binding events are rapid and saturable. After initial rapid Tau-EGCG interaction, equilibrium is attained where, constant association and dissociation of Tau-EGCG occurs. Similar nature of interaction has been reported for HSA-EGCG interaction where two separate binding events occur. There is initial strong binding between HSA and EGCG followed by 1,000 times weaker secondary binding event^34^. EGCG is thought to act via a common mechanism for various aggregation prone proteins.

**Table 1:** Parameters and calculated values for Tau-EGCG binding constant.

Titration ratio (Tau : EGCG)	1:25
Temperature (°C)	25.2
Bin	Binding
[Syringe]	500 μM
[Cell]	20 μM
N (sites)	3.48
Kd	74 nM
ΔH (kcal/mol)	− 1.04
ΔG (kcal/mol)	− 9.73
− TΔS (kcal/mol)	− 8.69

### Molecular models of Tau protein

In order to determine the putative binding site of EGCG to Tau and check if the hexapeptide repeat regions VQIINK and VQIVYK play any major role in the interactions between EGCG and Tau, a molecular model of Tau was generated. However, since Tau is a highly dynamic and disordered protein, predicting a model for its entire 441 residues is a near impossible task. Yet, the hexapeptide repeat regions are relatively structurally stable as corroborated by other previously reported biochemical and NMR data. So, a stretch of 147 amino acid residues including the hexapeptide regions and a few surrounding residues were used to make the model of Tau (Fig. 1E), which was used for further studies. These residues were finalized based on a BLAST search against PDB, which identified homologous structures, which could be used as templates to build the homology model of this region. NMR based structure of Tau (residue stretch 267–312) bound to microtubules (2MZ7) shared an identity of 100% and query coverage of 92% with the target sequence, and were hence used for modeling studies. The stereochemical parameters of the initial models were verified and it was observed that more than 98% of all residues were placed in the allowed regions of the Ramachandran plot. To further improve the model, refinement strategies involving preprocessing of the initial models (by adding hydrogens, assigning bond order, and filling missing loops and side chains) were performed. Following that, a restrained minimization was performed on the models, by applying constraints, which would serve to converge the non-hydrogen atoms to an RMSD of 0.3 using the OPLS 2005 force field. Finally, the models were subjected to 500 steps of steepest descent energy minimization, followed by 1,000 steps of conjugate gradient energy minimization using the same force field. These energy-minimized models were further used for docking and molecular dynamics studies.

### Molecular docking of repeat Tau and EGCG

**Figure 2:**
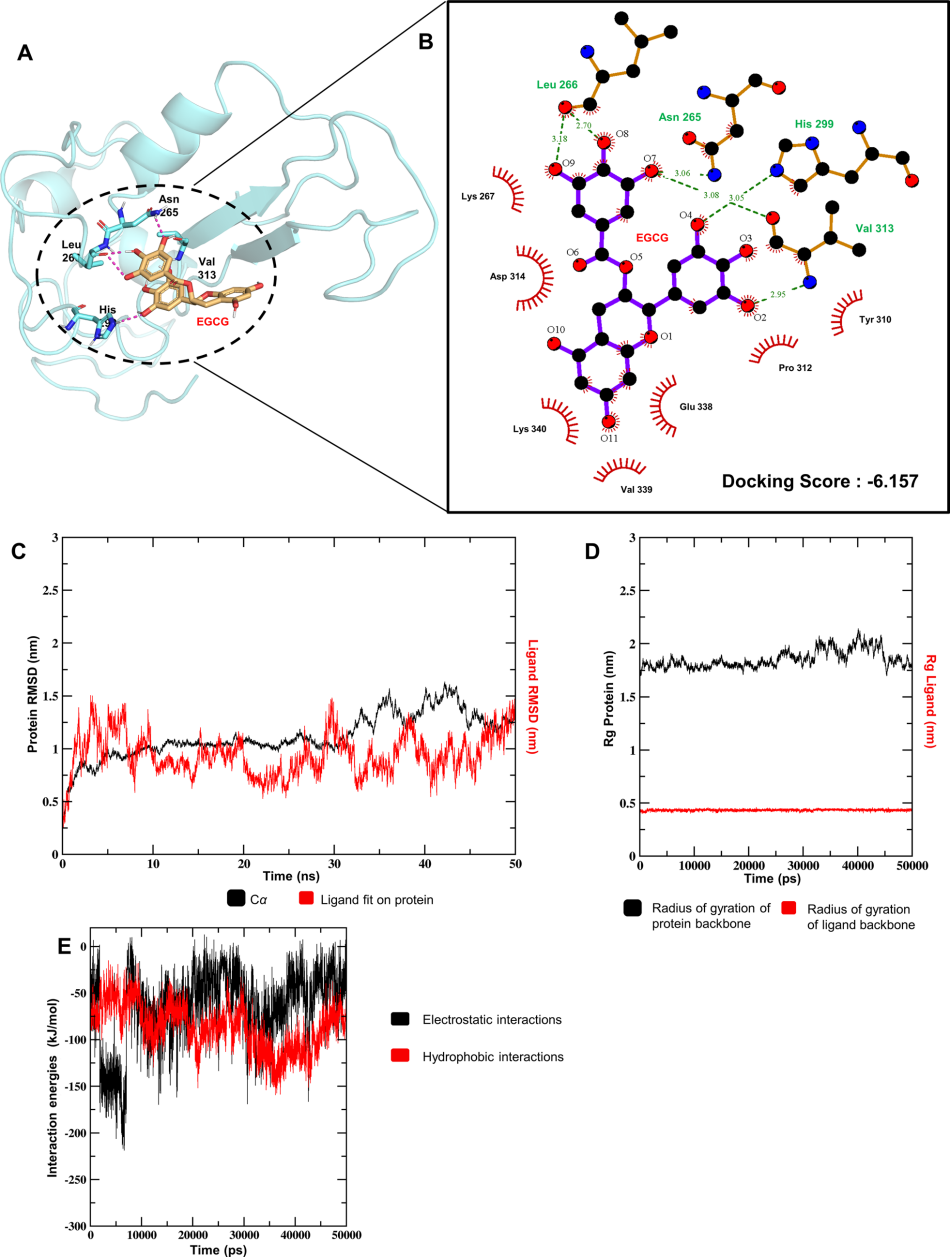
Simulation studies of Tau and EGCG. (**A**) The binding pocket of EGCG to the Tau model, shown along with the interacting residues after molecular docking. (**B**) The interaction between EGCG and Tau in the binding pocket is depicted, including the major residues involved and the predicted residues, which might be responsible in further interactions as well as the type of interactions they are involved in are shown. (**C**) The RMSD evolution of the protein (left Y-axis) and that of the ligand (right Y-axis) are shown. While the protein RMSD gives insights into the structural conformation of the protein throughout the simulation, the ligand RMSD indicates how stable the ligand is with respect to the protein and its binding pocket. Both the RMSDs are seen to stabilize as the simulation progresses. The ligand RMSD is notably stable of the two, suggesting that the binding pocket conformation is maintained throughout the simulation. (**D**) The Rg values of protein (left Y-axis) and ligand (right Y-axis) are shown. Both Rg values do not show much variation indicating that the structure of the protein and the binding pose of the ligand are maintained over the course of the simulation. (**E**) The electrostatic and hydrophobic interactions between the ligand and protein over the entire timescale of the simulation are depicted, showing the dominant, yet highly dynamic and transitionary nature of the same.

The docking studies of EGCG with repeat Tau model generated the best pose with glide score and E-model values of − 6.388 and − 56.130, respectively. EGCG was docked in a binding pocket in the vicinity of the predicted alpha helices and antiparallel beta sheets with the gallate moiety being in close proximity to the beta sheet and the Epigallocatechin backbone towards the helices (Fig. 2A). The interaction between EGCG and Tau repeats was majorly mediated by hydrogen bonding and hydrophobic interaction. The OH group involving O4 atom of EGCG interacts with the NH group of the imidazole ring of His 299. The OH groups involving the O2 and O7 atoms of EGCG interact with the NH_2_ group and carboxyl group of Val 313, respectively. The O7 atom also interacts with the NH_2_ group of the carboxamide group of Asn 265, while the OH groups involving the O8 and O9 atoms interact with the carboxyl O atom of Leu 266 forming hydrogen bonds in the process (Fig. 2A). The three OH groups (O7, O8 and O9) have interaction distances of 3.08 Å, 2.7 Å and 3.18 Å, respectively interacting with carbonyl groups. The interactions with the amine groups (by O2, O4 and O7) on the other hand, stand at interaction distances of 2.95 Å, 3.05 Å and 3.06 Å, respectively (Fig. 2B). Further a hydrophobic Pi-alkyl interaction was observed with residue Lys 340 while residues Asp 314 and Glu 338 are found to make a Pi-anion electrostatic interaction with benzene rings of the ligand EGCG. A few other residues Lys 267, Tyr 310, Pro 312 and Val 339 were also involved in hydrophobic interactions with the ligand. Interestingly, one of the interacting residues, Tyr 310 belongs to one of the hexapeptide repeat regions ^306^VQIVYK^311^ and the others are seen to majorly flank both the hexapeptide repeat regions (^275^VQIINK^280^ and ^306^VQIVYK^311^) (Fig. 1E), both of which are a prime area of interest in our study and thereby may be involved in the probable mechanism of Tau PHF dis-aggregation in presence of EGCG.

### Molecular dynamics simulation of repeat Tau and EGCG

The EGCG docked complex was used for a molecular dynamic simulation study of 50 ns time duration to examine the stability of the interaction and other relative structural changes, which could shed some light on the probable molecular events behind the binding of EGCG to Tau. The RMSD graphs plotted by assigning time in nanosecond (ns) on the X-axis and RMSD values of the Cα atoms of the protein and ligand in nanometers (nm), respectively on the left and right Y-axis (Fig. 2C), indicated that the RMSD of Cα atoms of the protein is relatively stable over the first 30 ns of the simulation, then undergoes slight fluctuation, only to re-stabilize in the last 5 ns. The fluctuation may be due to the partial destabilization of local bonds in the binding pocket of the protein due to the EGCG interaction. The ligand backbone over the complete trajectory showed that the system attains stability over short durations at different time points, which might be indicative of the characteristic binding and partial dissociation events showed by EGCG. As a result, we also observed some repositioning of the initial binding pose of EGCG with respect to the hexapeptide repeat region ^306^VQIVYK^311^ during the course of simulation. The Cα RMSD difference between the initial and final conformation after the simulation of docked complex is therefore ~ 0.75 Å which suggested some overall conformational change in Tau upon binding of EGCG as mentioned above and the system finally converged over the last few nanoseconds. The R_g_ (Radius of gyration) graph plotted by assigning time in picoseconds (ps) on the X-axis and R_g_ values of the protein and ligand backbones in (nm) on the left and right Y-axis, respectively (Fig. 2D) also indicated the compactness of the protein and ligand throughout the simulation. The interaction energy calculation between the representative residues and the ligand EGCG showed that both the Coulombic interactions were more prominent in the initial part of the simulation while Lennard-Jones interactions gain prominence as we move towards the end of the simulation, suggesting that the electrostatic and hydrophobic interactions of the Tau-EGCG complex are quite dynamic in nature (Fig. 2E). During the course of the simulation, residues Asn 265, Leu 266 and Val 313 majorly contribute to the hydrogen bond interactions. Other residues like Lys 267, Tyr 310, Pro 312 and Lys 340 contribute extensively to the hydrophobic interactions involving the protein and ligand. The residue specific interactions indicate that these may be the most crucial residues involved in the stabilization of the Tau-EGCG complex. We observed that the overall hydrogen bonding dynamics was maintained over the course of the simulation (Fig. S5). This included other ion-mediated and water mediated hydrogen bonding along with the residue specific interactions, which was maintained in the entire time duration of the simulation. These interactions might be the key to the dynamics between Tau and EGCG interaction.

To predict the binding site of EGCG with a Tau monomer and to gain insight into the molecular nature of the interaction of EGCG with Tau, we performed docking and simulation studies of the same. Tau protein having highly disordered N and C termini is structurally highly dynamic, thereby making it highly improbable to predict a reliable full-length Tau model. However, we have successfully obtained a reliable model for the regions of Tau surrounding the hexapeptide repeats from available structural data. Our results indicated that EGCG binds to one of these hexapeptide repeats. The nature of interaction is primarily hydrogen bonding, further stabilized by the water-mediated hydrogen bonds and hydrophobic interactions that led to a series of short-term interactions, binding and partial dissociation events throughout the simulation. Towards the end of the simulation timescale, hydrophobic interactions and probably ion or water-mediated hydrogen bonds are found to become more prevalent which suggests notable conformational changes of this aggregation prone region of Tau after EGCG binding.

### NMR spectroscopic studies of repeat Tau-EGCG interaction

**Figure 3:**
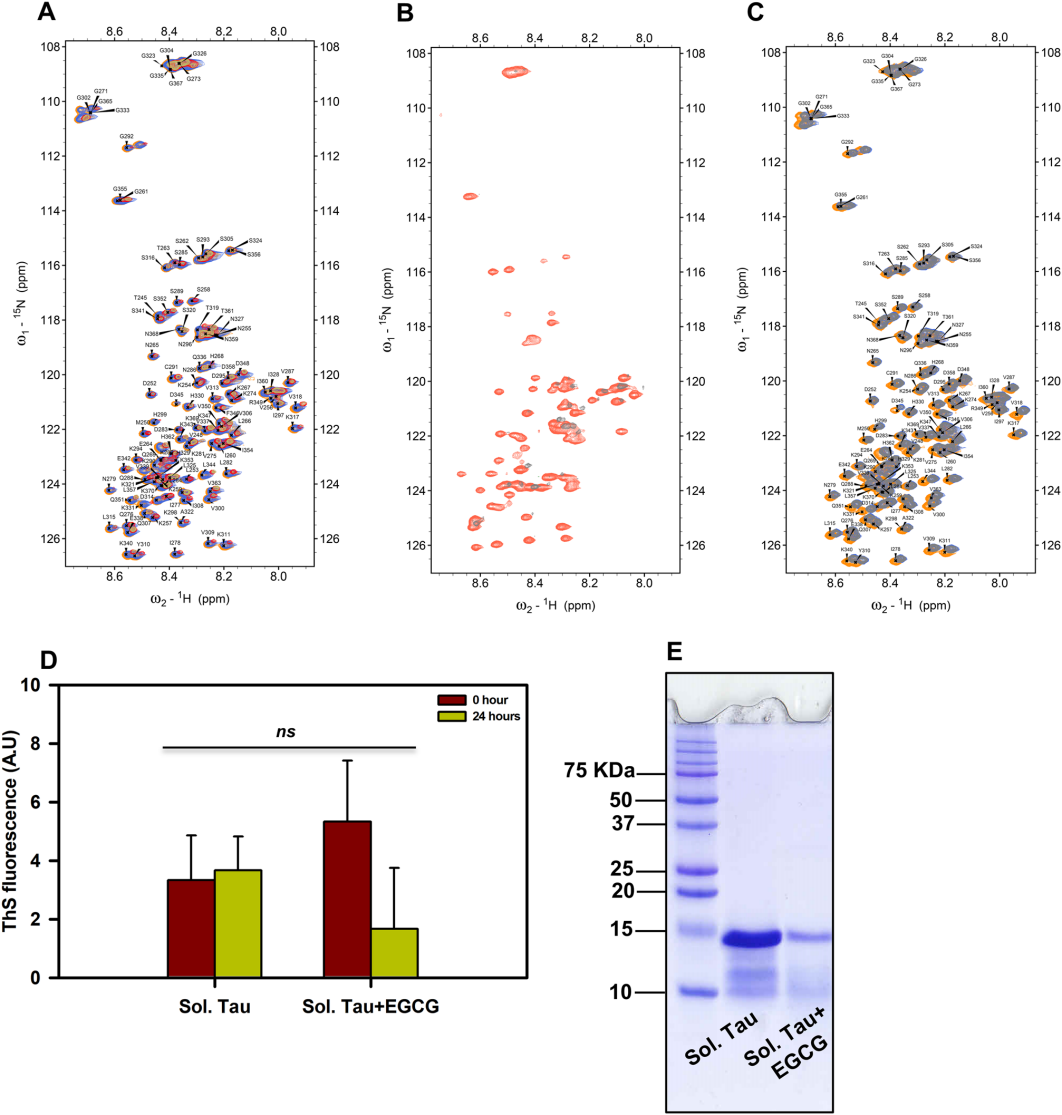
NMR spectroscopic studies of repeat Tau with EGCG. ^1^H-^15^ N-HSQC plots showing the chemical shift perturbations. For all the NMR experiments a solution of 200 μM of repeat Tau in 500 μL phosphate buffer containing 10% D_2_O is used. (**A**) Overlay of ^1^H–^15^N-HSQC plots (at 278 K) for the titration of 200 μM of repeat Tau against 0 μM (in orange), 100 μM (in blue), 500 μM (in red), 1,000 μM (in olive green) and 2000 μM (in coral, no visible signals) of EGCG. Continuous drop in the intensity of HSQC cross peaks indicates an increasing precipitation of repeat Tau with increasing concentrations of EGCG. (**B**) Overlay of ^1^H–^15^N-HSQC plot of repeat Tau at 298 K with that of repeat Tau in presence of 2000 μM of EGCG (sample from titration at 278 K, which was brought back to 298 K). (**C**) Overlay of ^1^H–^15^N-HSQC plot of repeat Tau (at 278 K) (without EGCG, golden) along with ^1^H–^15^N-HSQC plots measured for the precipitation of 200 μM of repeat Tau in presence of 500 μM of EGCG after t = 0 h (blue) and t = 24 h (grey). Upon addition of 500 μM of EGCG, the HSQC cross peaks are slightly displaced and lost intensity indicating precipitation of repeat Tau. After 24 h standing, no apparent change was observed in the HSQC cross peak intensity. (**D**) ThS fluorescence of NMR samples at 0 and 24 h suggesting no aggregation of repeat Tau in presence of EGCG. (**E**) SDS-PAGE analysis of NMR samples at 24 h showing no aggregation of repeat Tau in presence of EGCG.

Interaction between repeat Tau and EGCG was monitored by ^1^H–^15^N HSQC NMR spectroscopic studies using 200 μM of ^15^N labeled repeat Tau dissolved in phosphate buffer. First, repeat Tau was titrated with variable concentrations of EGCG at ratios 0, 0.5, 2.5, 5 and 10 with respect to 200 μM of repeat Tau at 278 K (Fig. 3A). The titration did not reveal any residue specific interaction of EGCG with repeat Tau and no significant conformational reorganization of repeat Tau. However, the intensities of HSQC cross peaks diminished with increasing amounts of EGCG until no signal was detected from the protein at 2000 μM of EGCG, which implies the precipitation of repeat Tau into larger molecular weight forms. Consistently, the contents of the NMR tube have turned turbid over the titration in support with the precipitation and loss of signal. After the titration, the final contents (200 μM of repeat Tau at 2000 μM of EGCG) of the NMR tube were brought to 298 K, allowed to stand for an hour and an HSQC spectrum was recorded (Fig. 3B, grey), which showed very weak signals and was superimposed on the HSQC of repeat Tau (without EGCG) at 298 K. These results indicated that a low concentration of precipitate solubilized back into monomeric repeat Tau, probably with native conformation, as the pattern of HSQC cross peaks of solubilized repeat Tau almost match with that of the native repeat Tau cross-peaks in HSQC. In order to investigate if the repeat Tau precipitate steadily increases over time at a given concentration of EGCG, we have carried out an experiment where ^1^H–^15^N HSQCs were acquired for 24 h at 278 K on a 200 μM repeat Tau sample in presence of 500 μM of EGCG (Fig. 3C). As seen from Fig. 3C, the HSQC after t = 0 h (blue) and t = 24 h (grey) are exactly superimposable, indicating no seeding effect of the precipitates at these concentrations. Further to confirm protein precipitation by EGCG, we monitored the ThS fluorescence of the sample from NMR tube at 0 time point and at the end of NMR titrations. It was observed that the precipitate did not give any increased fluorescence suggesting that protein actually precipitated and not aggregated (Fig. 3D). The sample at 24 h was also analyzed by SDS-PAGE and the EGCG sample did not show any higher order aggregates confirming our inference that EGCG precipitates repeat Tau protein (Fig. 3E).

### EGCG impedes human full-length Tau assembly

**Figure 4:**
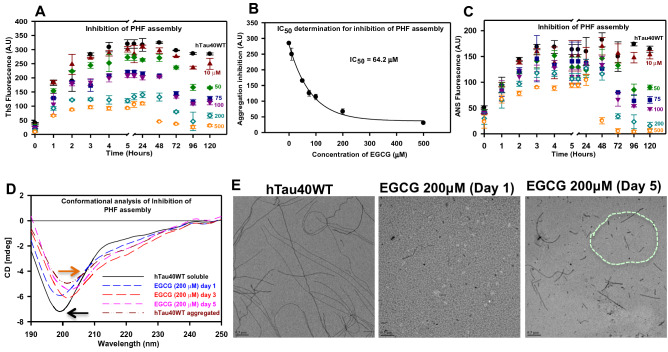
EGCG prevents Tau aggregation and changes their conformation in vitro. (**A**) The inhibitory effect of EGCG on the polymerization of full-length Tau monitored by ThS fluorescence. (**B**) The IC_50_ for EGCG for full-length Tau is 64.2 μM. (**C**) The inhibition of Tau PHF assembly assessed by ANS fluorescence shows a time and dose dependent decrease in the hydrophobicity of the Tau protein. (**D**) The CD analysis of EGCG treatment shows the formation of mixed Tau structures in a time dependent manner. (**E**) The control reaction shows the presence of long mature filaments whereas the EGCG treatment shows increase in the fragility of the Tau filaments at with increasing in time. The 200 μM EGCG treatment shows broken filaments at the day 5 of incubation.

Tau Paired Helical Filaments (PHFs) formation is induced in vitro by a positively charged anionic co-factor heparin. The effect of EGCG on this assembly was studied by adding a range of concentrations (10–500 μM) to the Tau (20 μM) assembly mixtures. The classical fluorescence assay with ThS demonstrated an aggregation impeding effect in presence of EGCG in a concentration-dependent manner. The aggregation was observed to progress till 24 h of incubation after which the fluorescence intensity reduced gradually as time advanced (Fig. 4A). This suggests that EGCG interferes with Tau PHFs assembly and shows 89% inhibition at highest concentration (500 μM) (Fig. S2A). The IC_50_ value for Tau assembly inhibition was determined to be 64.2 μM (Fig. 4B). In the course of PHF assembly, the hydrophobicity of aggregates increases. The effect of EGCG on the hydrophobicity changes during the PHF assembly was monitored by ANS fluorescence. ANS maps the hydrophobicity changes by emitting enhanced fluorescence as the hydrophobicity increases. ANS fluorescence was observed to increase as the initial aggregation of Tau proceeded in control and EGCG treated samples but was seen decreasing in the EGCG treated Tau in a concentration dependent manner with time (Fig. 4C). The percent inhibition with respect to ANS fluorescence was found to be 96% (Fig. S2B). Time dependent SDS-PAGE analysis of EGCG treated Tau was carried out to confirm effects of EGCG on Tau assembly. The 0 h SDS-PAGE profile confirmed the monomeric Tau in control and treated samples (Fig. S3A, 0 h) and absence of any preformed aggregates. At 6 and 12 h a tetramer band is observed in all the lanes suggesting progression of Tau assembly. However, at 24 h, the highest concentration of EGCG (500 μM) shows a complete loss of higher order Tau aggregates. This confirms with the fluorescence kinetics data, which drops after 24 h of incubation (Fig. S3A, 24 h). As the incubation advances all the treated samples show a drop in Tau aggregates on SDS-PAGE as well as its densitometric quantification (Fig. S3B). These results together demonstrate the role of EGCG in inhibiting Tau aggregation.

### EGCG causes a partial conformation change of Tau and increases the filament fragility

EGCG has been shown to prevent the formation of β-sheet structure by α-synuclein protein and rather help in adopting the random coil structure^35^. To check its effect on Tau conformation, we treated Tau with 1:10 ratio of EGCG and monitored the conformational changes in a time dependent manner. The aggregated Tau shifts its conformation from random coil to partial β-sheet (Fig. 4D). EGCG treated Tau also showed a partial β-sheet conformation in a time dependent manner. On the other hand, the aggregation kinetics showed an inhibition of PHFs assembly but after only after 24 h wherein basal level of intermediates must have formed. The partial β-sheet conformation might be due to these aggregate intermediates formed during early aggregation phase. The visualization of EGCG mediated inhibition of Tau assembly was carried out by TEM (Transmission Electron Microscopy) analysis. The untreated control showed long and abundant Tau PHFs whereas; the time dependent analysis of EGCG treated Tau showed only small pieces of Tau filaments at day 5 (Fig. 4E). The EGCG treated day 1 samples show almost absence of mature filaments (Fig. 4E). Thus, EGCG hampers the formation of mature Tau PHFs.

### EGCG is potent in dissolving preformed Tau filaments and oligomers

**Figure 5:**
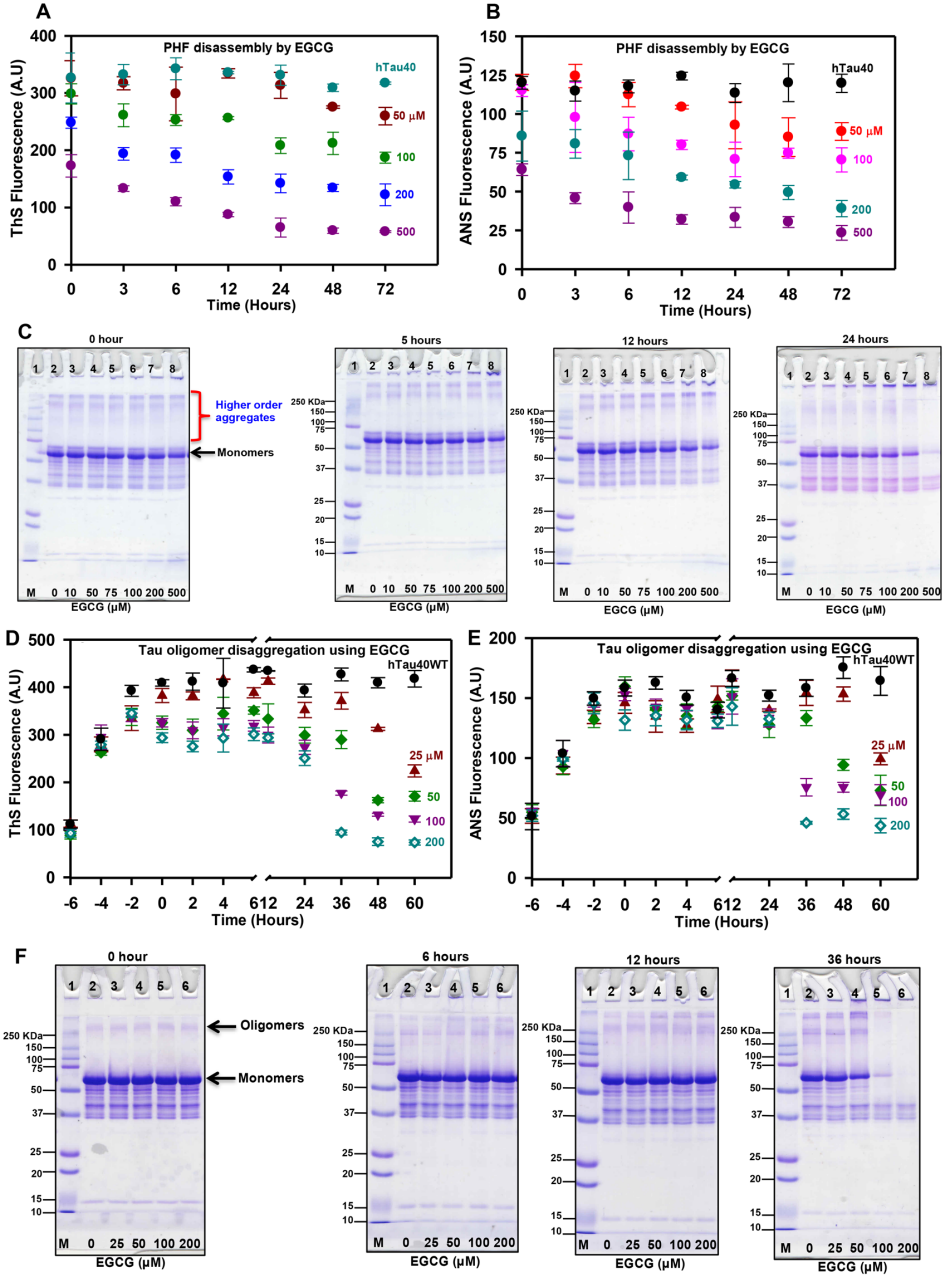
The preformed Tau fibrils and oligomers dissolved by EGCG. (**A**) The EGCG mediated dissolution of mature Tau fibrils recorded by ThS fluorophore showing the disassembly of PHFs. (**B**) The ANS fluorescence shows a time dependent decrease as the fibrils are dissolved. (**C**) The SDS-PAGE analysis of disassembly of Tau PHFs. (**D**) The ThS fluorescence shows the inhibitory effect of EGCG on the dissolution of Tau oligomers in a concentration dependent manner. (**E**) The ANS fluorescence shows the decrease in intensity with time and concentration suggesting loss of hydrophobicity of Tau oligomers. (**F**) The SDS-PAGE analysis shows the presence of oligomers at the time of compound addition (0 h) which are slowly cleared with time.

Aggregate formation is the key target in AD, but dissolution of the preformed aggregates also needs to be addressed as this aggregate load hampers neuronal function and gradually leads to neuronal death. Thus, EGCG was evaluated for its role in dissolving preformed Tau filaments. The ThS fluorescence kinetics demonstrated a drop in intensity in time dependent manner in EGCG treated reactions as opposed to control, which showed steady fluorescence throughout the incubation (Fig. 5A, S4A). The ANS fluorescence also showed a drop in intensity with time suggesting a decrease in hydrophobicity as the Tau aggregates were dissolved upon EGCG treatment (Fig. 5B, S4B). The SDS-PAGE analysis revealed the dissolution of aggregates in time and concentration dependent manner. The highest concentration of 500 μM showed complete clearance of Tau aggregates at 24 h (Fig. 5C, 24 h lane 8). EGCG is thus potent in inhibiting Tau PHF assembly as well as dissolving the preformed fibrils.

The toxicity of Tau oligomers is more pronounced than the mature aggregates. Tau oligomers were allowed to form till six hours after which EGCG was added to reaction mixtures at various concentrations (25–200 μM). This time point was considered as 0 h. Upon addition the kinetics was monitored at timed intervals. The highest concentration of 200 μM did not show increase in fluorescence from 0 h. The intensity remained stagnant till 12 h and dropped from 24 h onwards. This suggests EGCG captures oligomers and dissolves them and does not allow further aggregation (Fig. 5D, E, S4C, S4D). The oligomers before addition of EGCG were observed on SDS-PAGE (Fig. 5F, 0 h). The addition of EGCG gradually dissolved the oligomers in time dependent manner (Fig. 5F).

**Figure 6:**
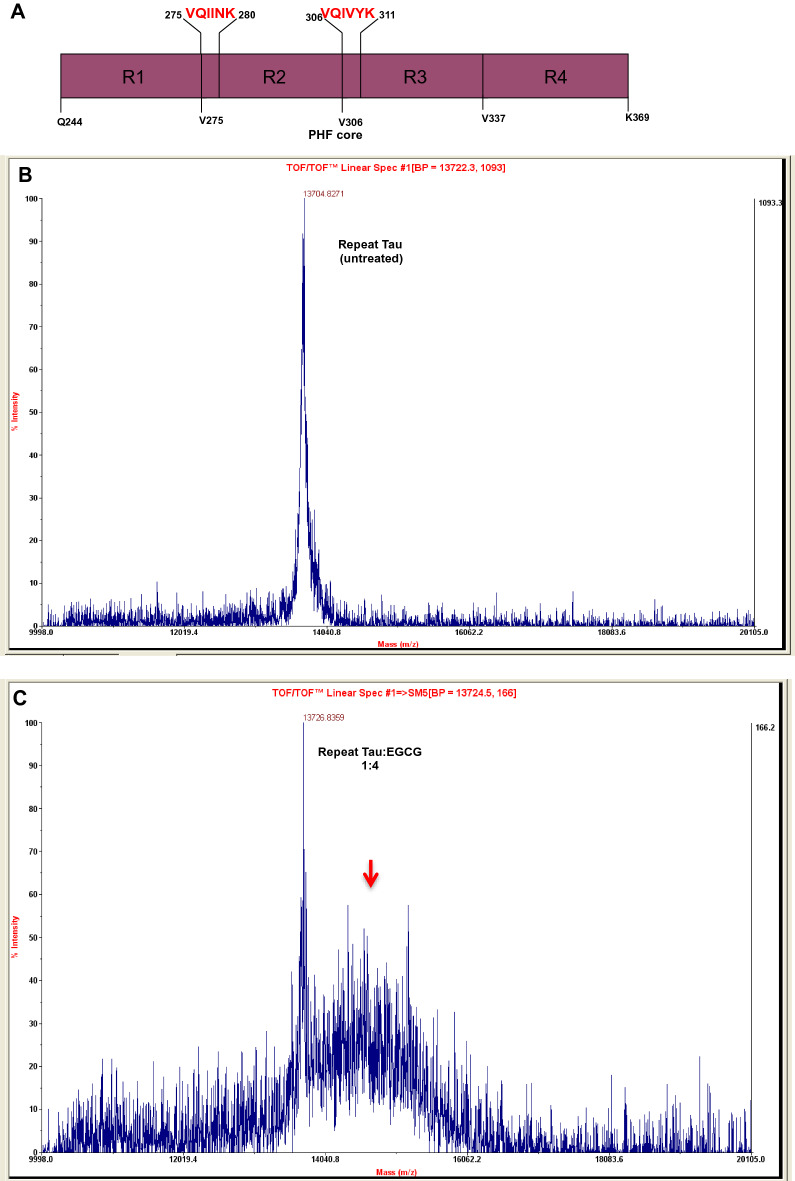
Modification of repeat Tau by EGCG. (**A**) Repeat domain of Tau showing two hexapeptide motifs. (**B**) The MALDI-TOF spectra of untreated repeat Tau showing a single peak at desired molecular weight of 13.7 KDa. (**C**) The EGCG treated repeat Tau shows an adjacent peak (red arrow) in addition to the soluble Tau peak suggesting the modification of Tau with EGCG as increase in molecular weight.

Since the in vitro and computational data suggested Tau-EGCG interaction and binding, we further performed MALDI-TOF analysis to check for the covalent modification of repeat Tau (Fig. 6A) by EGCG. EGCG treatment showed the adduct formation with repeat Tau at Tau: EGCG, 1:4 ratios, which can be seen as small peak (Fig. 6C, red arrow) in addition to the peak corresponding to soluble repeat Tau. The untreated control showed a single peak corresponding to 13.7 KDa for repeat Tau (Fig. 6B).

### Tau forms non-toxic higher order aggregates in presence of EGCG

**Figure 7:**
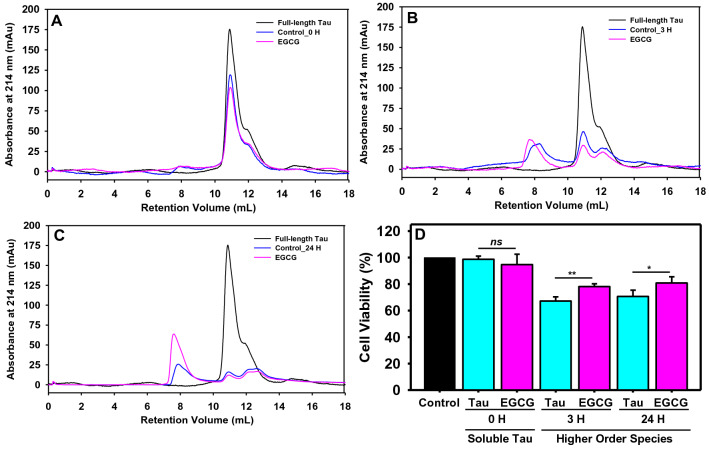
SEC for polyamines treated Tau. Tau was subjected to aggregation in presence of heparin as inducer. (**A**) At 0 h Tau was eluted as monomer, both in presence and absence of EGCG. (**B**) Further incubation led to aggregation of Tau, at 3 h of incubation EGCG showed more aggregation in Tau. (**C**) Similarly, at 24 the peak intensity of Tau in presence of EGCG was high when compared to control. This suggests that EGCG is driving Tau towards the formation of higher order aggregates. (**D**) Cell viability studies indicates that these conformers were non-toxic in neuro2A cells.

Tau forms a mixture of higher order species while aggregating which can be separated by SEC (Size Exclusive Chromatography). The full-length Tau was eluted as a monomer at 0 h (Fig. 7A, indicated in black). After 3 h of incubation Tau was eluted as two distinct peaks at retention volumes 8.3 and 7.7 mL for control and EGCG, respectively (Fig. 7B), indicating the formation of higher order species. Further at 24, control as well as EGCG treated Tau eluted at the retention volume of 7.6 mL (Fig. 7C as a single peak indicating the higher order aggregates, Table 2). However, the early species formed at 3 h of incubation clearly demonstrates that EGCG accelerates the formation of Tau aggregates when compared to Tau alone. Neuroblastoma cells treated with the fractions obtained from SEC did not show significant toxicity (Fig. 7D).

**Table 2:** Peak retention volumes for EGCG treated and untreated Tau.

Time (h)	Tau ± EGCG	Rentention volume (mL)
0	Soluble Tau	10.9
	Control	10.9
	EGCG	10.9
3	Control	8.3
	EGCG	7.7
24	Control	7.9
	EGCG	7.6

### EGCG enhances neuronal growth

**Figure 8:**
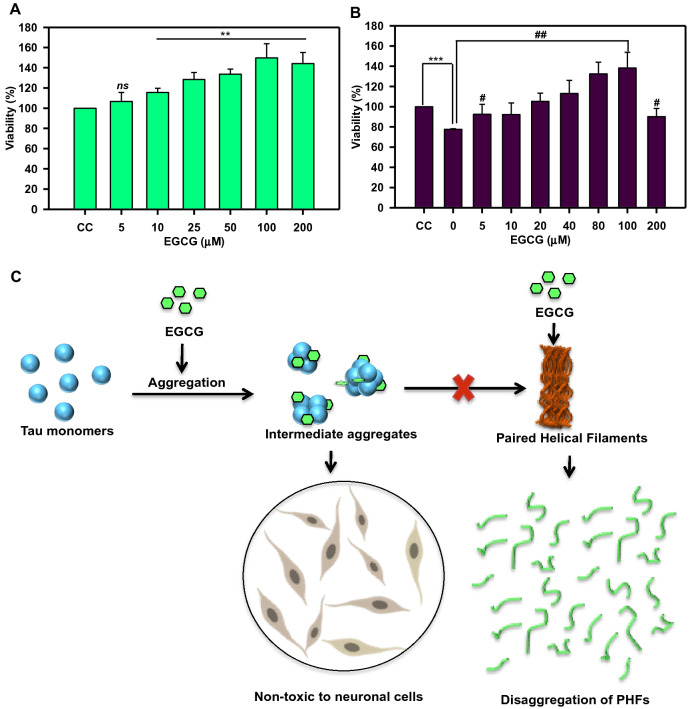
Effect of EGCG on cell viability. Toxicity was induced with 5 μM Tau aggregates and rescue in presence of EGCG (0–200 μM) in neuro2a cells. (**A**) Cell viability assay shows EGCG was non-toxic to neuronal cells at varying concentrations (0–200 μM) p < 0.001 and enhance the cell survival. (**B**) Tau aggregates induce toxicity in neuro2a cells at 0 μM EGCG p < 0.05(denoted as *) EGCG significantly rescues toxicity of Tau aggregate mediated toxicity till and enhances viability p < 0.05 (denoted as #). (**C**) EGCG inhibits Tau aggregation by forming intermediate Tau aggregates and disintegrating them. The intermediate aggregates formed are non-toxic to neuronal cells. On the other hand, when matured Tau fibrils are treated with EGCG, they are disaggregated and cleared.

EGCG is a known neuroprotective and antioxidant molecule^27^. Its effect on neuro2a cell viability revealed that EGCG is not only non-toxic but it also enhances cell survival with varying concentrations (Fig. 8A). Neuro2a cells treated with Tau aggregates (5 μM) and 0 μM of EGCG showed 77% viability as compared to control. When cells were treated with aggregates along with EGCG, a significant rescue of toxicity was observed along with enhanced survival (Fig. 8B).

## Discussion

Tau aggregation inhibitors in Alzheimer’s disease have yielded numerous hits, which are potent in alleviating the Tau pathology. Several natural compounds are gaining much attention as effective therapeutics against Tau pathology^36,37^. The molecular targets^38,39^ of these molecules vary since Tau undergoes a series of intermediate species formation before leading to mature PHFs. We have screened EGCG for its efficacy against full-length human Tau aggregation. EGCG has already been reported to inhibit in vitro aggregation of a mutant repeat Tau (K18ΔK280). However, the heparin induced aggregation of the Tau fragment K18ΔK280 could not be rescued by EGCG^33^. Moreover, this gave rise to Tau filaments that are morphologically different from the untreated filaments. In our study, we demonstrate the inhibition of heparin induced full-length Tau aggregation by EGCG with altered filament morphology and number. The IC_50_ for inhibition is higher as compared to other molecules and this might be due to the fact that heparin induction cuts down on the lag phase, which might be essential for efficient binding of EGCG to monomeric Tau for impeding the Tau assembly. Thus, EGCG might need to bind to the intermediate species to inhibit aggregation in large stoichiometric ratios. EGCG has been implied in covalently modifying proteins at the reduced Cys thiols. Our MALDI-TOF results suggests that EGCG forms adducts with repeat Tau thus explaining the presence of a higher order band in nitrobluetetrazolium (NBT) staining from the previous studies on EGCG and repeat Tau^33^. Along with mechanism of action, the exact interaction of EGCG with the misfolding prone proteins have being studied. Although the number and nature of interacting amino acid residues vary according to proteins, some key interacting residues remain common for such proteins. In EGCG-Tau docking studies we have found Leu and Asp residues showing major interactions, which were also reported to be the interacting residues in transthyretin complex^40^. One of the major contributors in the interactions between Tau and EGCG was found to be Lysine 267 and it has been reported previously, that a lysine residue in prostatic acid phosphatase (PAP) peptide was found to have a strong interaction with EGCG, and was an important residue in ligand binding. ITC results suggested binding between Tau and EGCG occurs in a multi-sites binding event such that the initial binding events are rapid and saturable. After initial rapid Tau-EGCG interaction, equilibrium is attained where, constant association and dissociation of Tau-EGCG occurs. Similar nature of interaction has been reported for HSA-EGCG interaction where two separate binding events occur. There is initial strong binding between HSA and EGCG followed by 1,000 times weaker secondary binding event^34.^ EGCG is thought to act via a common mechanism for various aggregation prone proteins. It is speculated that EGCG binds to the cross-beta sheet intermediates and prevents further assembly^41^. Our study demonstrated this effect for full-length Tau wherein an initial oligomerization was visualized with SDS-PAGE and later a gradual decrease in the oligomers. On the other hand, the disassembly of Tau PHFs was very rapid. This might be attributed to the already present aggregation intermediates allowing rapid binding and action of EGCG. In titration of repeat Tau with EGCG, NMR spectroscopic studies have shown clear loss of repeat Tau signal intensity with increasing concentrations of EGCG. The repeat Tau signals have all vanished at 1:10 concentration ratio of repeat Tau to EGCG in concurrence with the precipitation observed in the NMR tube. The precipitate from the NMR tube was collected and characterized by ThS. Very poor intensity in ThS fluorescence assay for the precipitate indicates that its morphology is different from regular heparin induced aggregates of Tau or repeat Tau.

The dual role of EGCG in disassembling Tau filaments and quenching the oligomers makes it a potent candidate for future drug designing.

## Conclusion

EGCG interacts with full-length Tau at multiple residues with unstable interactions. It inhibits aggregation of full-length Tau and dissolves Tau fibrils and oligomers. We predict that EGCG forms higher order structures and degrades them without allowing the formation of mature aggregates, which was also observed in NMR analysis. The higher order structures formed by EGCG are non-toxic to neuroblastoma cells and EGCG rescues the aggregate mediated toxicity in the neuroblastoma cells (Fig. 8C). Thus, EGCG acts to inhibit Tau aggregation by direct but unstable interactions.

## Materials and methods

### Chemicals and reagents

Luria–Bertani broth (Himedia); Ampicillin, Heparin, NaCl, Phenylmethylsulfonylfluoride (PMSF), MgCl_2,_ Sodium azide, APS, DMSO, Ethanol (Mol Bio grade), Chloroform, Isopropanol (Mol Bio grade) were purchased from MP biomedicals; IPTG and Dithiothreitol (DTT) from Calbiochem; EGCG, ThS, ANS, Glycine, MES, BES, SDS, MTT from Sigma; EGTA, Tris base, 40% Acrylamide, TEMED from Invitrogen. Protease inhibitor cocktail was purchased from Roche. For cell culture studies, Dulbecco modified eagle’s media (DMEM), Fetal bovine Serum (FBS), Phosphate buffer saline (PBS, cell biology grade), Trypsin–EDTA, Penicillin–streptomycin were purchased from Invitrogen.

### Tau purification

Protein purification for full-length Tau was carried out as previously described^42^. In brief, full-length recombinant Tau was expressed in *E.coli* BL21* strain. The cell pellets were homogenized under high pressure (15,000 psi) in a microfluidics device for 15 min. The obtained lysate was heated at 90 °C for 15 min after addition of 0.5 M NaCl and 5 mM DTT. The heated lysate was then cooled and centrifuged at 40,000 rpm for 50 min. The supernatant was collected and dialyzed in Sepharose A buffer overnight. The obtained dialyzed sample was then subjected to a second round of ultracentrifugation and the supernatant was loaded onto the cation exchange column (Sepharose fast flow GE healthcare) for further purification. The bound protein was eluted using an ionic gradient. The eluted proteins were pooled and concentrated for size exclusion chromatography (16/600 Superdex 75 pg GE healthcare). The Tau concentration was measured using BCA method^42^.

### Molecular modelling

To procure templates for homology model building, a similarity search using Basic Local Alignment Search Tool (BLAST) algorithm was performed against the Protein Data Bank (PDB), to identify high-resolution crystal structures of homologous proteins^43,44^. The sequence identity cut off was set to ≥ 30% (E-value cut off = 1). Homology modeling of Tau K18 was then carried out using Modeller 9.16 by taking structures homologous to the target proteins as templates, in order to study their structural features, binding mode and affinity with the substrates.

### Model validation and refinement

The initial models obtained, were evaluated for the stereochemical quality of the protein backbone and side chains using PROCHECK and RAMPAGE^45^. ERRAT server checked the environments of the atoms in the protein model. Errors in the model structures were also checked with ProSA server^46,47^. After model validation, initial models were refined using impref minimization of protein preparation wizard and Impact 5.8 minimization. These energy minimized final models were further used for the binding studies with their substrates.

### Ligand–protein preparation and docking studies

The ligand molecule considered in the present study was downloaded from ZINC compound database in the mol2 format. The protein and ligands were prepared first before proceeding with the docking studies. The water molecules and other heteroatom groups were removed from the protein structures using protein preparation utility of Maestro. Hydrogens were added subsequently to carry out restrained minimization of the models. The minimization was done using impref utility of Maestro in which the heavy atoms were restrained such that the strains generated upon protonation could be relieved. The root mean square deviation (RMSD) of the atomic displacement for terminating the minimization was set as 0.3 Å. Similarly, ligands were refined with the help of LigPrep 2.5 to define their charged state and enumerate their stereoisomers. The processed receptors and ligands were further used for the docking studies using Glide 5.8^48^. Sitemap analysis was performed on the prepared receptor molecule, to identify the probable binding sites for our ligands of interest, since no prior information was available regarding the same^49^. Next, grids were generated by selecting any of the Sitemap points obtained above. Flexible ligand docking was carried out using the standard precision option. A total of 6 poses with the respective ligand and different sites were generated and scored on the basis of their docking score, glide score and E-model values. The hydrogen bond interactions between the protein and ligands were visualized using PyMOL.

### Molecular dynamics simulations

The docked complex with the lowest Glide score and Glide E-model values were used to perform molecular dynamics simulation using the GROningen MAchine for Chemical Simulations V4.5.4 (GROMACS)^50,51^ with the CHARMM36 force field. The docked complex was placed in the center of a dodecahedron box solvated in water. The ligand topology files and the other force field parameter files pertaining to the ligand were created using the official CHARMM General Force Field server CGenFF^52,53^. The SPC216 water model was used and the distance between the solute and the box was set to 10 Å. The dimensions of the initial simulation cell were kept at approximately 90 × 90 × 90 Å. for the simulation and the initial energy minimization of the system was carried out by steepest descent minimization for 50,000 steps, till a tolerance of 10 kJ/mol was attained, to make sure that the high energy interactions and steric clashes in the system could be avoided during simulation. A suitable number of Cl^−^ ions were added to balance the total negative charges on the docked structures to make the whole system neutral using the genion program of GROMACS and the system was again subjected to energy minimization by steepest descent minimization retaining the same parameters. The system was stabilized at 300 K temperature and pressure of 1 bar using the Vrescale, a modified Berendsen thermostat, temperature coupling^54^ and Parrinello-Rahman pressure coupling methods^55^. The Partial Mesh Ewald (PME) algorithm^56^ was employed for computing electrostatic and van der Waals interactions. A cut off distance of 9 Å and 14 Å was set for Coulomb and van der Waals interactions, respectively. The LINCS algorithm^57^ was used to apply rotational constraint to all the bonds. No positional constraints were applied on the system. Periodic boundary conditions were applied in all three directions. The complex in the medium was equilibrated for 100 ps in *NPT* and *NVT* ensembles. Finally, a 50 ns molecular dynamics simulation was carried out for the protein–ligand complex and all trajectories were stored every 2 ps for further analysis. The trajectories were visualized using Visual Molecular Dynamics program (VMD)^58^. The energies and RMSD of the complex in each trajectory were monitored with respect to simulation time. The intermolecular interactions between the target and substrate were assessed to check the stability of the complexes. The R_g_ (Radius of gyration) was also monitored for the protein and ligand backbones to check their stability in the active site pocket as well as their overall compactness.

### NMR spectroscopy

For NMR experiments, a 200 μM solution of ^15^N labelled repeat Tau protein was prepared in 10:90 D_2_O:H_2_O aqueous medium of 50 mM phosphate buffer also containing 1 mM DTT. ^1^H–^15^N HSQC experiments have been acquired using 512 increments and 2 k complex points in the indirect and direct dimension, respectively, and 16 scans per increment. The data was processed by Bruker topspin software and analyzed using Sparky. NMR experiments for titration and aggregation of repeat Tau with EGCG were acquired at 278 K on Bruker Avance III HD 700 MHz spectrometer equipped with a TXI probe. Re-dissolving of repeat Tau precipitates (obtained in presence of EGCG) was monitored by acquiring the ^1^H–^15^N HSQC experiments at 298 K.

### Isothermal titration calorimetry

Isothermal titration calorimetry was carried out for full-length Tau and EGCG in 1:25 ratio in a PEAQ-ITC micro calorimeter at 25 °C. Full-length Tau (20 μM) was titrated with EGCG (500 μM) EGCG in sodium phosphate buffer, pH 7.4. Titration experiment comprised of a total of 25 injections of 1.5 μl for 3 s at an interval of 180 s between each injection. An initial injection of 0.4 μl was given for stabilization of reaction cell. The data was analyzed and interpreted using PEAQ-ITC software using one set of sites model. The final baseline corrected data and heat plot were plotted using Sigma plot 10.2. The values of ΔG, ΔH and TΔS were used to calculate the binding constant for the interaction.

### Tau aggregation assay

The soluble human Tau40wt protein was centrifuged at 60,000 rpm for 1 h (Optima Max XP Beckman Coulter) to ensure presence of monomeric Tau and 20 μM of soluble Tau protein was incubated in 20 mM BES buffer pH 7.4 with 5 μM of Heparin 17,500 Da in presence of 25 mM NaCl 1 mM DTT protease inhibitor cocktail, 0.01% Sodium azide, and different concentrations of EGCG ranging from 0 to 500 μM. EGCG was dissolved in ultrapure water and stored in 4 °C. Aggregation inhibition and disaggregation studies were carried out in presence of 1 mM DTT as a reducing agent. The reaction mixtures were incubated at 37 °C. The aggregation was studied by ThS and ANS fluorescence assay.

### ThS fluorescence

The Tau protein in the reaction mixture and ThS were incubated in ammonium acetate pH 7.0 at concentrations of 2 μM and 8 μM, respectively for 10 min. The readings were taken by exciting ThS at 440 nm and collecting the emission data at 521 nm in TECAN Infinite M 200 pro. The buffer measurements were subtracted from the sample readings before plotting the data.

### ANS fluorescence

For ANS measurements the Tau and ANS were incubated at 2 μM and 40 μM, respectively in ammonium acetate pH 7.0 for 20 min. The fluorescence measurements were taken at excitation/emission 390 nm/475 nm in TECAN Infinite M 200 pro. The buffer blank were subtracted and the readings were taken in triplicates.

### Tau disassembly assay

Tau filaments were prepared to a concentration of 100 μM in the assembly buffer mentioned above for 8 days at 37 °C. After confirmation of the mature filament formation by ThS fluorescence and SDS-PAGE, these were diluted to 20 μM and with were treated EGCG (0 to 500 µM) at 37 °C. Further, the dissolution of Tau fibrils was monitored by fluorescence assays and SDS-PAGE.

### Circular Dichroism Spectroscopy

CD spectroscopy was carried out to check effect of EGCG on Tau aggregation inhibition on Jasco J-815 CD spectrometer. The sample was diluted to 3 μM Tau in sodium phosphate buffer pH 6.8 in a 1 mm cuvette. The bandwidth and scan speed were set as 1 nm and 100 nm/min, respectively. The scan was carried out from 250 to 190 nm with an average of 5 acquisitions. Buffer baseline was subtracted from each reading.

### Electron microscopy

The qualitative analysis for Tau aggregation inhibition and disassembly by EGCG was performed by Transmission electron microscopy. The 400 mesh carbon coated copper grids were inubated with 2 μM of the reaction 45 s. The grids were given 2 washes of filtered MilliQ. Further, the grids were incubated with 2% for 1 min and dried. The scanning was performed on Tecnai G2 20 S-Twin transmission electron microscope.

### MALDI-TOF analysis

Repeat Tau protein (1 mg/ml) was incubated with EGCG for 12 h at 37 °C. The samples were then diluted 1:20 in Sinapic acid and spotted on the MALDI plate and analyzed using ABSCIEX 4800 MALDI-TOF analyzer.

### Size Exclusion Chromatography (SEC)

20 µM of full-length Tau was incubated in assembly buffer in presence and absence of 100 µM EGCG. Tau was subjected to size-exclusion using Superdex 200 increase at 0, 3, 24 h. The change in retention volume was recorded against time of incubation. The intensity of Tau with and without EGCG at each time point in correspondence to retention volume was plotted in terms of bar. The respective fractions obtained after SEC were analysed for their toxicity in neuro2a cells. The SEC experiments were carried out in AKTA Pure M (GE Healthcare).

### Cell viability assay

10,000 neuro2a cells were seeded in 96 well culture plate. The cells were incubated at 37 °C, 5% CO_2_. Cells were treated with the fractions for 24 h. Post-treatment cells were treated with 0.5 mg/mL of MTT(3-(4,5-dimethylthiazol-2-yl)-2,5-diphenyltetrazolium bromide) and incubated for 4 h. Thus formed formazone crystals were dissolved in 100 µL of DMSO. This leads to the formation of purple coloured complex whose absorbance was measured at 590 nm in TECAN Infinite Series Pro 200 spectrofluorimeter.

### Statistical analysis

All the statistical analyses were carried out using unpaired T-test by SigmaPlot 10. The error bars represent mean ± SD values. 95% confidence intervals were maintained for the analyses.

### NMR accession number

Repeat Tau chemical shift assignments deposited in BMRB (accession number: 19253) have been used for the analysis.

## Supplementary information

Supplementary file1 (PDF 770 kb)

## Abbreviations

EGCG: Epigallocatechin-3-gallate
AD: Alzheimer’s disease
Aβ: Amyloid-β
IAPP: Islet amyloid polypeptide
ITC: Isothermal titration calorimetry
TEM: Transmission electron microscopy
SEC: Size exclusion chromatography
MTT: 3-(4,5-Dimethylthiazol-2-yl)-2,5-diphenyltetrazolium bromide
ThS: Thioflavin S
ANS: 8-Anilino-1-naphthalenesulfonic acid ammonium salt
