# Supplementary material for: EGCG impedes human Tau aggregation and interacts with Tau
Source: Sci Rep. 2020 Jul 28;10:12579. doi: 10.1038/s41598-020-69429-6 (PMC7387440; doi:10.1038/s41598-020-69429-6)
Supplement: Supplementary file 1 — Supplementary file1 (PDF 770 kb) [file 41598_2020_69429_MOESM1_ESM.pdf]

Supplementary Information

EGCG impedes human Tau aggregation and interacts with Tau

Shweta Kishor Sonawane[1,4], Hariharakrishnan Chidambaram[1,4], Debjyoti Boral[2,4], Nalini Vijay Gorantla[1,4,#], Abhishek Ankur Balmik[1,4,#], Abha Dangi[3,4], Sureshkumar Ramasamy[2,4], Udaya Kiran Marelli[3,4] and Subashchandrabose Chinnathambi[1,4,*]

[1]Neurobiology Group, Division of Biochemical Sciences, CSIR-National Chemical Laboratory, Dr. Homi Bhabha Road, 411008 Pune, India

[2]Structural Biology Group, Division of Biochemical Sciences, CSIR-National Chemical Laboratory, Dr. Homi Bhabha Road, 411008 Pune, India

[3]Central NMR Facility and Division of Organic Chemistry, CSIR-National Chemical Laboratory, Dr. Homi Bhabha Road, 411008 Pune, India

[4]Academy of Scientific and Innovative Research (AcSIR),411008 Pune, India

[#] Equal authors

*To whom correspondence should be addressed: **Prof. Subashchandrabose Chinnathambi,** Neurobiology group, Division of Biochemical Sciences, CSIR-National Chemical Laboratory, Dr. Homi Bhabha Road, 411008 Pune, India, Telephone: +91-20-25902232, Fax. +91-20-25902648. Email: s.chinnathambi@ncl.res.in

Supplementary figure 1

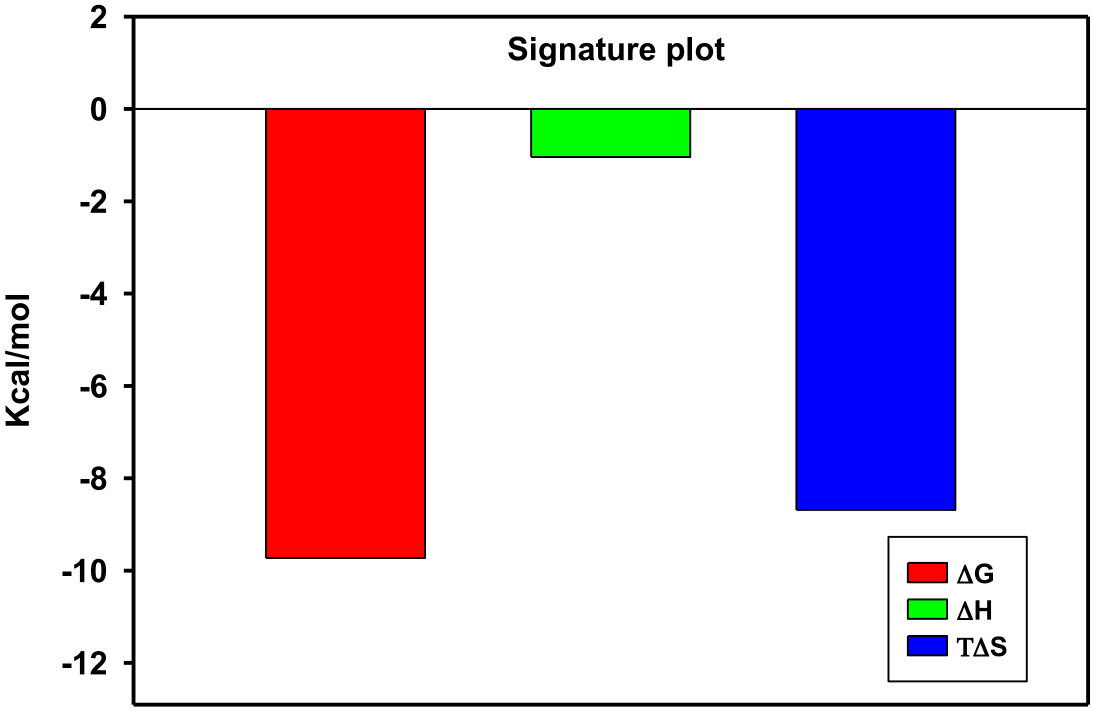

Figure S1. Signature plot for Tau-EGCG titration showing ΔG, ΔH and TΔS for the interaction.

Supplementary figure 2

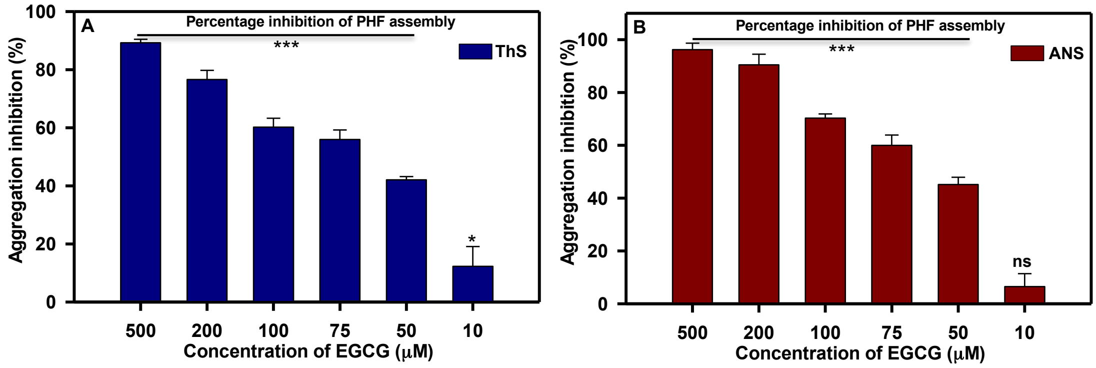

Figure S2. The quantification of Tau aggregation inhibition by EGCG: A) The percent inhibition of EGCG monitored by ThS shows a good 89% inhibition of fibrillization $p<0.001$. B) The ANS fluorescence shows 96% inhibition of Tau assembly $p<0.001$.

Supplementary figure 3

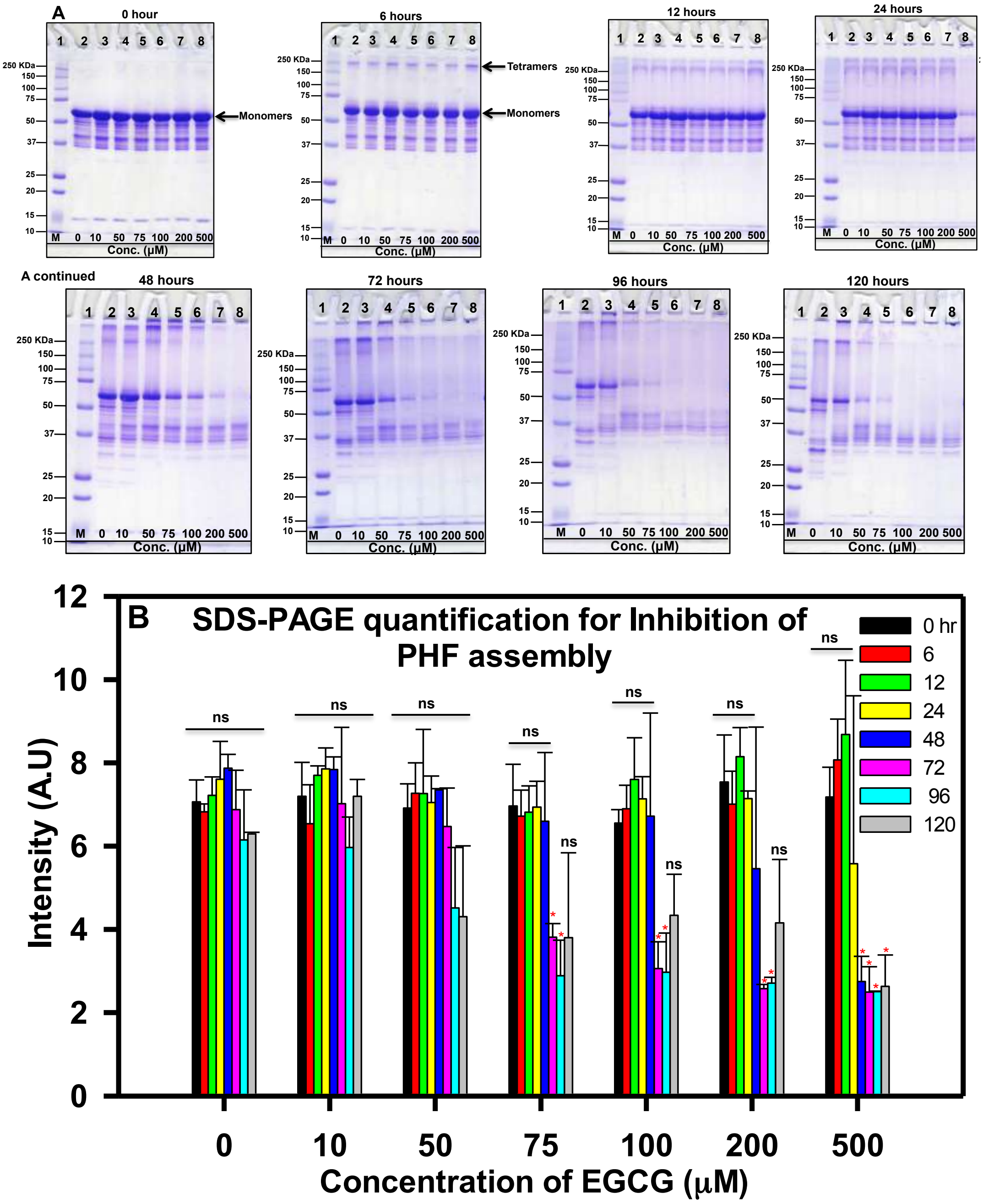

Figure S3. The effect EGCG on the formation of the SDS-resistant Tau aggregates. A) The analysis of control and EGCG treated samples on the SDS-PAGE shows initial formation of SDS-resistant tetramers at 6 hours of incubation that are slowly abolished in a time and concentration dependent manner. B) The densitometry analysis showing decrease in Tau aggregates $p<0.05$.

Supplementary figure 4

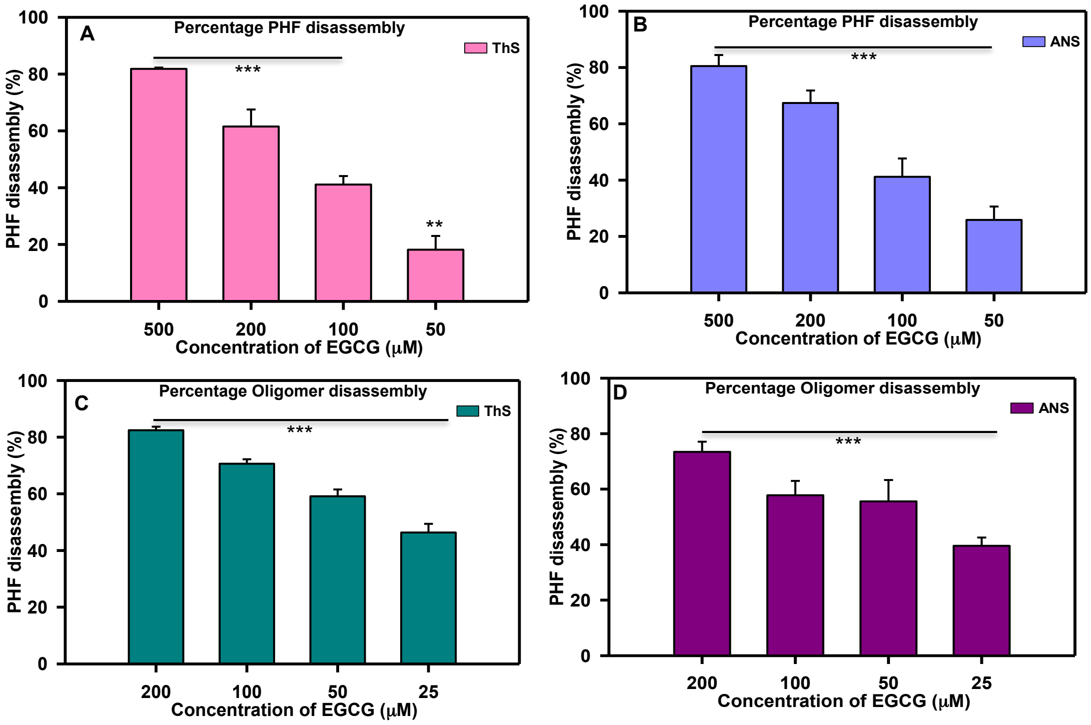

Figure S4. The quantification of preformed Tau fibrils dissolved by EGCG: A) The percent PHF disaasembly by EGCG monitored by ThS fluorescence showed 81% disassembly at highest concentration (500 μM) $p<0.001$. B) The ANS fluorescence shows 80% Tau fibril disassembly $p<0.001$. C) Tau oligomer disassembly was 82% by ThS fluorescence at 500 μM ($p<0.001$) D) ANS assay showed 75% of Tau oligomer disassembly at 500 μM $p<0.001$.

Supplementary figure 5

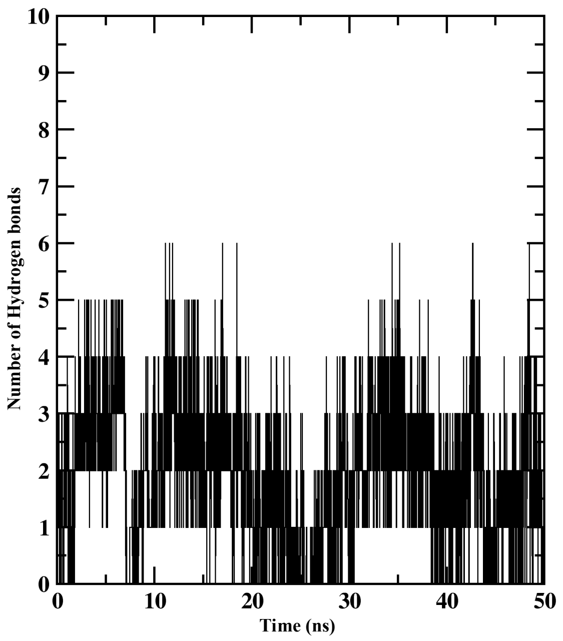

Figure S5. Hydrogen bond profile of Tau-EGCG complex simulation. The number of hydrogen bonds formed between Tau-EGCG complex over the entire duration of 50 ns simulation.

Supplementary Information

EGCG impedes human Tau aggregation and interacts with Tau

Shweta Kishor Sonawane[1,4], Hariharakrishnan Chidambaram[1,4], Debjyoti Boral[2,4], Nalini Vijay Gorantla[1,4,#], Abhishek Ankur Balmik[1,4,#], Abha Dangi[3,4], Sureshkumar Ramasamy[2,4], Udaya Kiran Marelli[3,4] and Subashchandrabose Chinnathambi[1,4,*]

[1]Neurobiology Group, Division of Biochemical Sciences, CSIR-National Chemical Laboratory, Dr. Homi Bhabha Road, 411008 Pune, India

[2]Structural Biology Group, Division of Biochemical Sciences, CSIR-National Chemical Laboratory, Dr. Homi Bhabha Road, 411008 Pune, India

[3]Central NMR Facility and Division of Organic Chemistry, CSIR-National Chemical Laboratory, Dr. Homi Bhabha Road, 411008 Pune, India

[4]Academy of Scientific and Innovative Research (AcSIR),411008 Pune, India

[#] Equal authors

*To whom correspondence should be addressed: **Prof. Subashchandrabose Chinnathambi,** Neurobiology group, Division of Biochemical Sciences, CSIR-National Chemical Laboratory, Dr. Homi Bhabha Road, 411008 Pune, India, Telephone: +91-20-25902232, Fax. +91-20-25902648. Email: s.chinnathambi@ncl.res.in

SDS-PAGE raw gels:

Figure 3

E

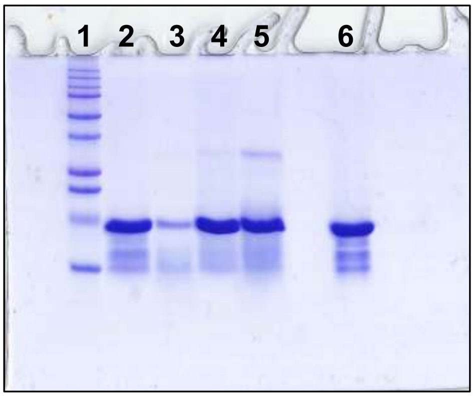

1. **Marker**
2. **Soluble repeat Tau**
3. **Soluble Tau+EGCG**
4. **Soluble Tau+heparin**
5. **Soluble Tau+heparin+EGCG**
6. **Soluble repeat Tau**

All the reactions were maintained at 5 °C for 24 hours

Figure 5

C

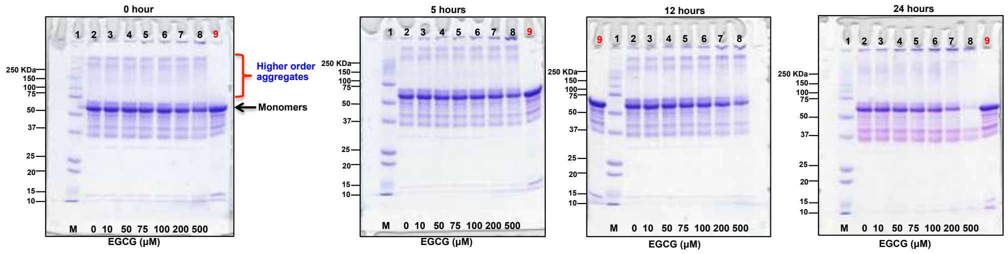

Above are the uncut SDS-PAGE for figure 5C in the main figures. The cropped lane number 9 corresponds to 20 µM soluble hTau40WT which was loaded as control at each time point.

F

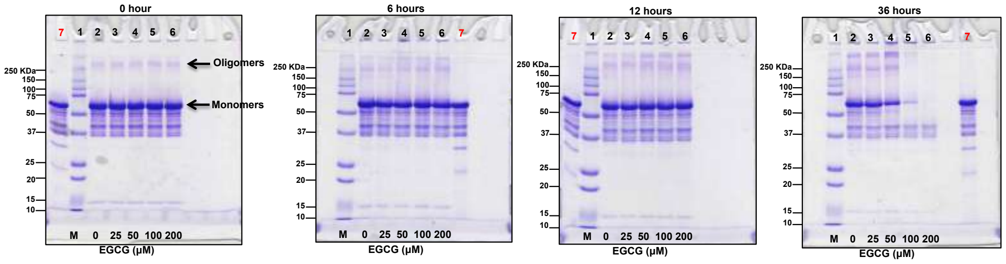

Above are the uncut SDS-PAGE for figure 5F in the main figures. The cropped lane number 7 corresponds to 20 µM soluble hTau40WT which was loaded as control at each time point. The rest of the wells are empty.

Supplementary figure 3

A

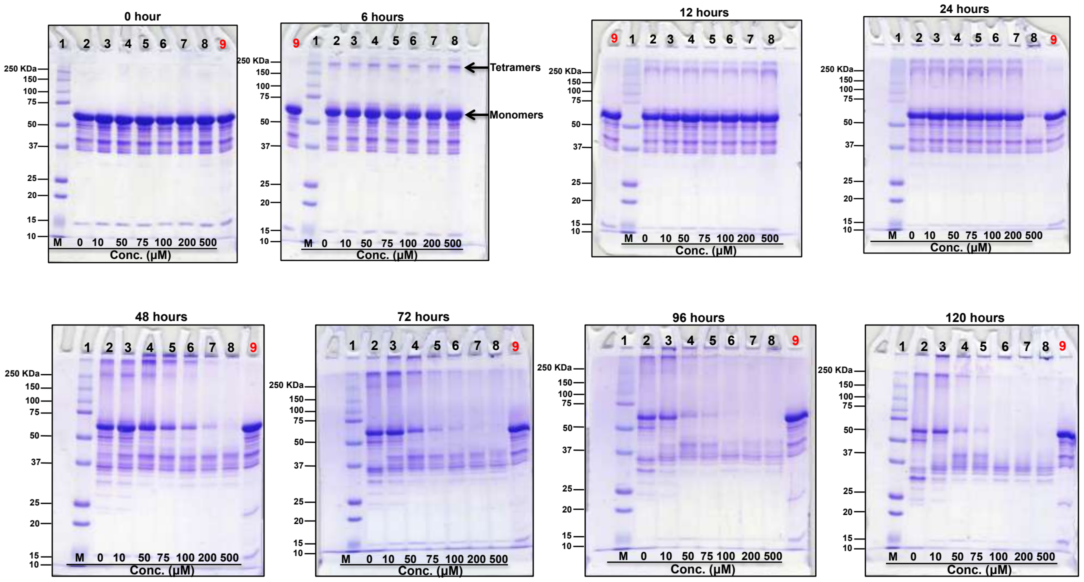

Above are the uncut SDS-PAGE for figure 5C in the main figures. The cropped lane number 9 corresponds to 20 µM soluble hTau40WT which was loaded as control at each time point.

Supplementary Information

EGCG impedes human Tau aggregation and interacts with Tau

Shweta Kishor Sonawane[1,4], Hariharakrishnan Chidambaram[1,4], Debjyoti Boral[2,4], Nalini Vijay Gorantla[1,4,#], Abhishek Ankur Balmik[1,4,#], Abha Dangi[3,4], Sureshkumar Ramasamy[2,4], Udaya Kiran Marelli[3,4] and Subashchandrabose Chinnathambi[1,4,*]

[1]Neurobiology Group, Division of Biochemical Sciences, CSIR-National Chemical Laboratory, Dr. Homi Bhabha Road, 411008 Pune, India

[2]Structural Biology Group, Division of Biochemical Sciences, CSIR-National Chemical Laboratory, Dr. Homi Bhabha Road, 411008 Pune, India

[3]Central NMR Facility and Division of Organic Chemistry, CSIR-National Chemical Laboratory, Dr. Homi Bhabha Road, 411008 Pune, India

[4]Academy of Scientific and Innovative Research (AcSIR),411008 Pune, India

[#] Equal authors

*To whom correspondence should be addressed: **Prof. Subashchandrabose Chinnathambi,** Neurobiology group, Division of Biochemical Sciences, CSIR-National Chemical Laboratory, Dr. Homi Bhabha Road, 411008 Pune, India, Telephone: +91-20-25902232, Fax. +91-20-25902648. Email: s.chinnathambi@ncl.res.in

Ancillary information

Ancillary 1

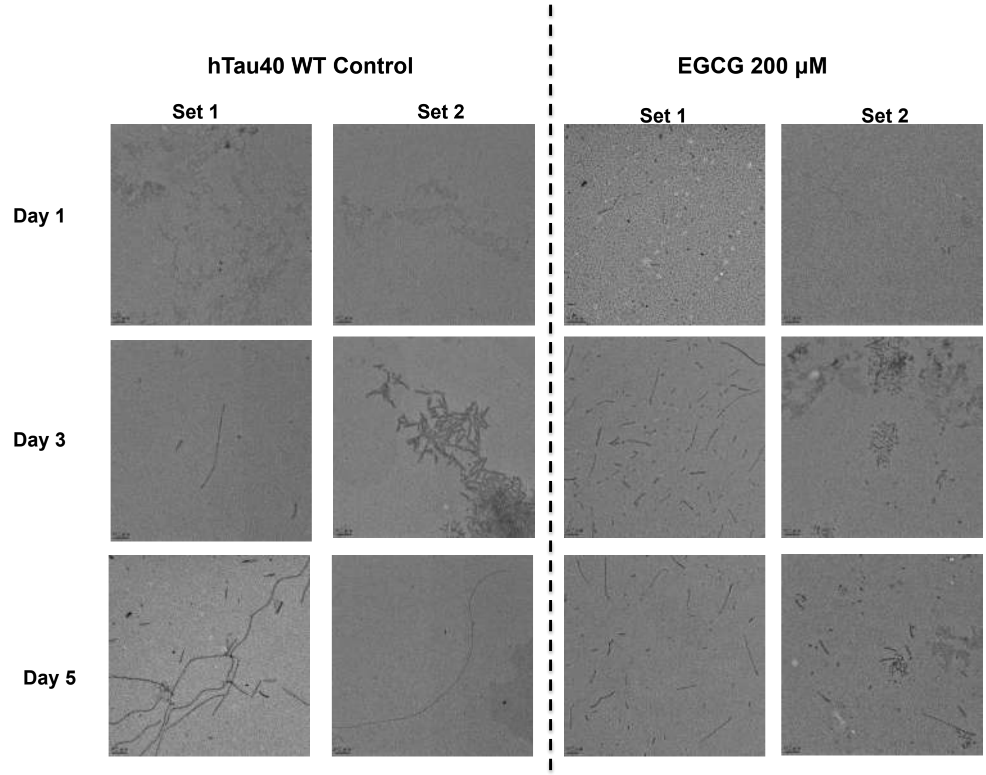

Ancillary 2

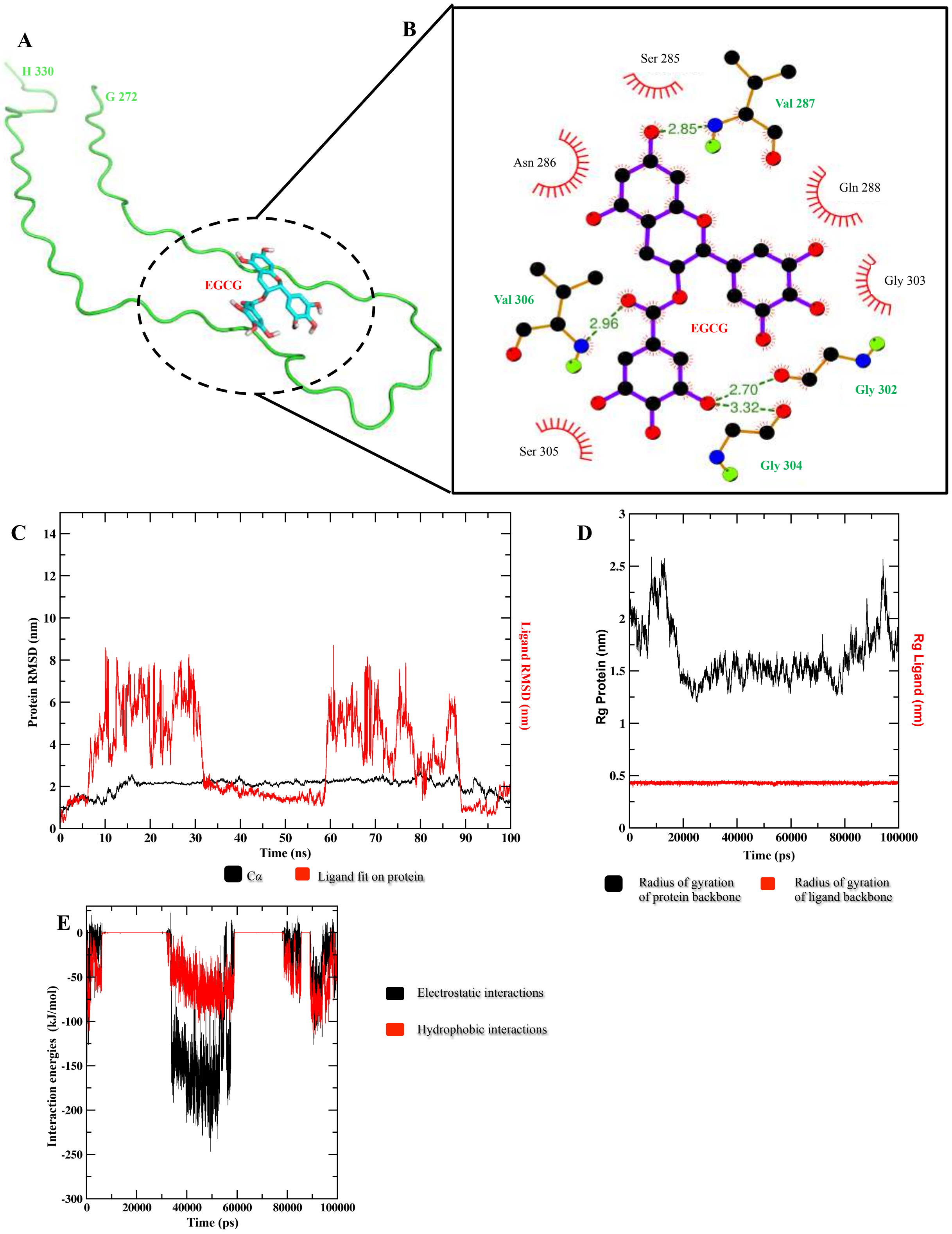

A
H 330
G 272
EGCG
B
Ser 285
Val 287
2.85
Asn 286
Gln 288
Gly 303
Val 306
2.96
EGCG
2.70
3.32
Gly 302
Ser 305
Gly 304
C
Protein RMSD (nm)
Ligand RMSD (nm)
Time (ns)
Cα
Ligand fit on protein
D
Rg Protein (nm)
Rg Ligand (nm)
Time (ps)
Radius of gyration of protein backbone
Radius of gyration of ligand backbone
E
Interaction energies (kJ/mol)
Time (ps)
Electrostatic interactions
Hydrophobic interactions

Ancillary 3

Kd values in respective runs

ITC run	Kd1	Kd2
**ITC Run on 210818**	4.91E-08	5.23E-12
**ITC Run on 08032020**	1.46E-09	1.70E-09
**ITC Run on 09032020**	1.31E-09	1.32E-09
**ITC Run on 11032020**	9.80E-09	1.06E-08

The ITC heat plots were analyzed by fitting into two set of sites model as per the previous study by Joshua D. Eaton *et al., 2017* for binding of EGCG and human serum albumin. The recent runs performed did not give proper fitting by any of the possible fitting models. But at the same time the binding constant range remained unchanged.

Run No. 1 (08032020)

20 µM Tau vs. 500 µM EGCG
Phosphate buffer pH 7.4 100 mM NaCl

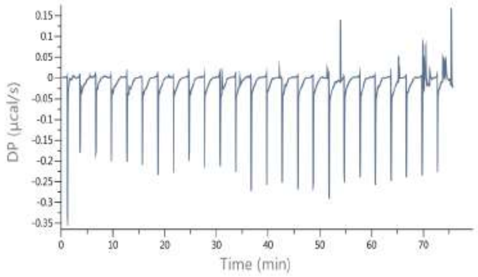

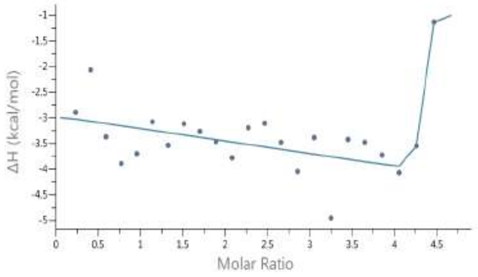

Two set of sites binding model

**Filename**	**20 µM hTau40wt 500 µM EGCG pH 7.4 100mM NaCl Run No. 1**
[Syr] (M)	5.00E-04
[Cell] (M)	2.00E-05
N_1 (sites)	2.58
KD_1 (M)	1.46E-09
ΔH_1 (kcal/mol)	3.2
ΔG_1 (kcal/mol)	-12.1
$-T\Delta S_1$ (kcal/mol)	-15.3
N_2 (sites)	1.65
KD_2 (M)	1.70E-09
ΔH_2 (kcal/mol)	-11.4
ΔG_2 (kcal/mol)	-12
$-T\Delta S_2$ (kcal/mol)	-0.533

Run No. 2
(09032020)

20 µM Tau vs. 500 µM EGCG
Phosphate buffer pH 7.4 100 mM NaCl

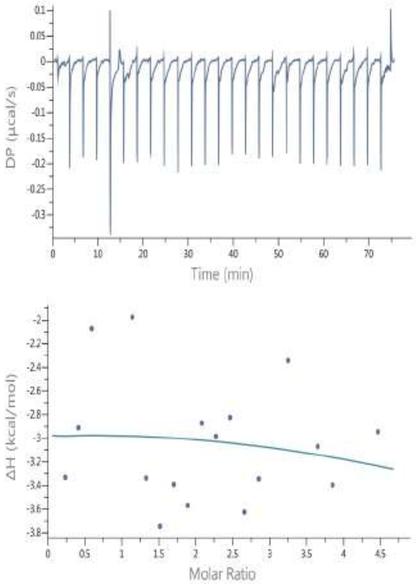

Two set of sites binding model

**Filename**	**20 µM hTau40wt 500 µM EGCG pH 7.4 100mM NaCl Run No. 2**
[Syr] (M)	5.00E-04
[Cell] (M)	2.00E-05
N_1 (sites)	5.76
KD_1 (M)	1.31E-09
ΔH_1 (kcal/mol)	-23.8
ΔG_1 (kcal/mol)	-12.1
$-T\Delta S_1$ (kcal/mol)	11.7
N_2 (sites)	6.08
KD_2 (M)	1.32E-09
ΔH_2 (kcal/mol)	74.3
ΔG_2 (kcal/mol)	-12.1
$-T\Delta S_2$ (kcal/mol)	-86.4

Run No.3
(11032020)

20 µM Tau vs. 500 µM EGCG
Phosphate buffer pH 7.4 100 mM NaCl

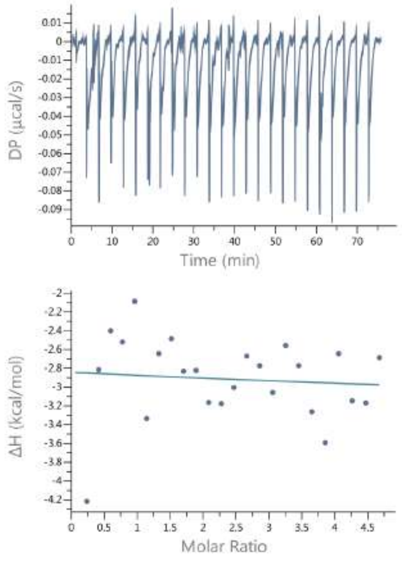

Two set of sites binding model

**Filename**	**20 µM hTau40wt 500 µM EGCG pH 7.4 100mM NaCl Run No. 3**
[Syr] (M)	5.00E-04
[Cell] (M)	2.00E-05
N_1 (sites)	4.42
KD_1 (M)	9.80E-09
ΔH_1 (kcal/mol)	1.24
ΔG_1 (kcal/mol)	-10.9
$-T\Delta S_1$ (kcal/mol)	-12.2
N_2 (sites)	3.18
KD_2 (M)	1.06E-08
ΔH_2 (kcal/mol)	-6.06
ΔG_2 (kcal/mol)	-10.9
$-T\Delta S_2$ (kcal/mol)	-4.83
